# Volatile Organic Compounds Produced by Human Pathogenic Fungi Are Toxic to *Drosophila melanogaster*

**DOI:** 10.3389/ffunb.2020.629510

**Published:** 2021-01-18

**Authors:** Hadeel S. Almaliki, Astrid Angela, Nayab J. Goraya, Guohua Yin, Joan W. Bennett

**Affiliations:** ^1^Department of Plant Biology, Rutgers, The State University of New Jersey, New Brunswick, NJ, United States; ^2^Technical Institute of Samawa, Al-Furat Al-Awsat Technical University, Samawa, Iraq

**Keywords:** *Aspergillus fumigatus*, *Candida albicans*, *Cryptococcus neoformans*, *Cryptococcus gattii*, *Drosophila melanogaster*, Virulence, volatile organic compounds

## Abstract

Volatile organic compounds (VOCs) are low molecular mass organic compounds that easily evaporate at room temperature. Fungi produce diverse mixtures of VOCs, some of which may contribute to “sick building syndrome,” and which have been shown to be toxigenic in a variety of laboratory bioassays. We hypothesized that VOCs from medically important fungi might be similarly toxigenic and tested strains of *Aspergillus fumigatus, Candida albicans, Cryptococcus neoformans, Cryptococcus gattii*, and *Saccharomyces cerevisiae* in a *Drosophila melanogaster* eclosion bioassay. Fungi were grown in a shared microhabitat with third instar larvae of *D. melanogaster* such that there was no physical contact between flies and fungi. As the flies went through metamorphosis, the numbers of larvae, pupae, and adults were counted daily for 15 days. After 8 days, ~80% of controls had eclosed into adults and after 15 days the controls yielded 96–97% eclosion. In contrast, eclosion rates at 8 days were below 70% for flies exposed to VOCs from six different *A. fumigatus* strains; the eclosion rate at 15 days was only 58% for flies exposed to VOCs from *A. fumigatus* strain SRRC 1607. When flies were grown in a shared atmosphere with VOCs from *S. cerevisiae*, after 15 days, 82% of flies had eclosed into adults. Exposure to the VOCs from the medically important yeasts C*andida albicans, Cryptococcus neoformans*, and *Cryptococcus gattii* caused significant delays in metamorphosis with eclosion rates of 58% for *Candida albicans*, 44% for *Cryptococcus neoformans*, and 56% for *Cryptococcus gattii*. Using gas chromatography-mass spectrometry, the VOCs from the most toxic and least toxic strains of *A. fumigatus* were assayed. The two most common VOCs produced by both strains were 1-octen-3-ol and isopentyl alcohol; however, these compounds were produced in 10-fold higher concentrations by the more toxic strain. Our research demonstrates that gas phase compounds emitted by fungal pathogens may have been overlooked as contributing to the pathogenicity of medically important fungi and therefore deserve more scrutiny by the medical mycology research community.

## Introduction

Volatile organic compounds (VOCs) are low molecular mass organic substances that are easily vaporized at room temperature (Herrmann, 2010). The best-known VOCs are synthetic chemicals used as solvents, cleaning agents and so forth; many of these industrial VOCs have toxigenic effects and their emissions have been subject to government regulation (Bennett and Inamdar, 2015). Less is known, however, about the toxigenic potential of biogenic VOCs emitted by fungi and other organisms as part of their normal metabolism. Fungal VOCs are released as mixtures of chemical compounds; different species growing on different substrates produce unique mixtures of VOCs (Korpi et al., 2009; Hung et al., 2015). VOC “signatures” provide rapid, inexpensive, and non-destructive indicators for recognizing the presence of agricultural or indoor mold contamination (Gao et al., 2002; Polizzi et al., 2009; Cabral, 2010; Pennerman et al., 2016). Similarly, researchers have sought to diagnose invasive aspergillosis by the detection of VOCs emitted in breath when the fungus is growing on the human host (Heddergott et al., 2014; Licht and Grasemann, 2020; Martínez et al., 2020). Fungal VOCs often have odors similar or identical to toxic industrial compounds and have been associated with symptoms of poor health such as headaches, dizziness, faintness, and irritation of the eyes and mucous membranes of the nose and throat (Takigawa et al., 2009; Araki et al., 2010, 2012). Many researchers have hypothesized that fungal VOCs have negative effects on human health with reference to processes like composting (Herr et al., 2003). In particular, it is thought that these VOCs may contribute to the symptoms of a poorly understood health condition called “sick building syndrome” (Mølhave et al., 1993; Hodgson, 1999; Heseltine and Rosen, 2009; Mølhave, 2009; Hosseini et al., 2020; Zuo et al., 2020).

Relatively little is known about the biological activity of VOCs associated with human fungal pathogens. *Aspergillus fumigatus*, for example, is not only an opportunistic human pathogen (Agarwal, 2009; Latgé and Steinbach, 2009) but it has been isolated from buildings whose occupants have complained of building-related illness as well as from the homes of asthmatic children (Schwab and Straus, 2004). *Candida albicans*, another opportunistic human pathogen, has been isolated as one of the contaminants present in hospital air (Pantoja et al., 2016). Known volatiles emitted by *A. fumigatus* and *C. albicans* include ethanol, acetaldehyde, acetone, methanethiol, 2-butenal, isoamyl alcohol, phenethyl alcohol, and cyclohexane as determined by ion mobility spectrometry and selected ion flow tube-mass spectrometry (SIFT-MS) (Scotter et al., 2005; Perl et al., 2011).

Our laboratory has adapted *D. melanogaster* as a model for studying the toxigenic potential of fungal volatiles. Flies are well-suited for toxigenic studies because the fly immune system is highly conserved with mammals. Furthermore, immune deficient flies have been used as models for studying aspergillosis and other human mycoses (Lionakis and Kontoyiannis, 2012). Here, we used an eclosion assay (the emergence of the adult insect from the pupal case during metamorphosis) that did not involve direct contact between fungi and flies (Inamdar et al., 2013; Rand et al., 2014). Third instar larvae of *Drosophila* were placed in a common atmosphere with VOCs produced by six different *A. fumigatus* strains, as well as one strain each of *Candida albicans, Cryptococcus neoformans, Cryptococcus gattii*, and *Saccharomyces cerevisiae* as a non-pathogen control. The numbers of larvae, pupae, and adults were counted daily for 15 days in order to determine effects of VOCs on the developmental process and/or eclosion into adults. Using a somewhat different version of this fly toxicity test, previous experiments in our laboratory showed that VOCs emitted by molds isolated from contaminated building materials after Hurricane Katrina and Superstorm Sandy caused developmental defects and death (Inamdar and Bennett, 2015; Zhao et al., 2017). Here we hypothesized that volatiles emitted by medically important fungi would have similar toxigenic properties. The long-term goal of our research is to learn whether VOCs emitted by fungi have physiological effects that can contribute to the severity of human disease. The specific aim of this study was to use the *Drosophila* larval bioassay to test the volatiles produced by medically important fungi to determine if they had toxicological impacts on fly metamorphosis.

## Materials and Methods

### Fungal Strains, Culture Conditions, Measurement of Biomass/Dry Weight

*A. fumigatus* strains were obtained from Dr. Geromy Moore, Southern Regional Research Laboratories, U. S. Department of Agriculture, New Orleans, Louisiana, USA. The *A. fumigatus* strain numbers and their original sources were listed in Table 1. Stock cultures were maintained on potato dextrose agar (PDA, Difco). For all *Drosophila* exposure experiments, the fungi were grown on 25 mL of PDA in 6 oz. *Drosophila* stock bottles (Genesee Scientific, CA). When fungi serve as human pathogens, they grow at 37°C. However, *Drosophila* flies do not grow at this temperature. Therefore, fungi were pre-grown for 3 days at 37°C, but when the fungal colonies were affixed to the bottles containing fly larvae, the 15-day exposure experiment was conducted at 25°C. Parallel experiments were conducted with fungi pre-grown at 25°C for 5 days in order to determine if fungal growth temperature was associated with different VOC profiles and associated toxicological effects. In order to determine if there was a difference in biomass, different strains of *A. fumigatus* grown at 25°C for 5 days or at 37°C for 3 days were compared. Spore suspensions of the different *A. fumigatus* strains were inoculated into 50 mL of Potato Dextrose Broth (PDB) and were incubated at 120 rpm at 25°C for 5 days or at 37°C for 3 days. The resultant mycelial pellets were filtered using Whatman filter paper No. 1 and dried at 50°C for 4 days in an air incubator to measure the biomass/dry weight of fungus (Singh et al., 2012). Three replicates were performed for each fungal strain and repeated two times.

**Table 1:** The details about toxicity and the origins of different fungal strains used in this study.

**Species**	**Origins**	VOCs toxicity ranking^*^
*Cryptococcus neoformans* H99	Serotype A, Public Health Research Institute Center, New Jersey Medical School-Rutgers, Newark, NJ, USA	10
*Cryptococcus gattii* R265	Wild type, Public Health Research Institute Center, New Jersey Medical School-Rutgers, Newark, NJ, USA	9
*Aspergillus fumigatus* SRRC1607	Damp indoor environment	8
*Candida albicans* ATCC 90028	Wild type, Public Health Research Institute Center, New Jersey Medical School-Rutgers, Newark, NJ, USA	7
*Aspergillus fumigatus* SRRC46	A penguin at the Brookfield Zoo, Chicago, IL	6
*Aspergillus fumigatus* SRRC323	Chicken lung	5
*Aspergillus fumigatus* SRRC2569	Clinical isolate from the University of Manchester, Manchester, UK	4
*Saccharomyces cerevisiae* BY4741	Public Health Research Institute Center, New Jersey Medical School-Rutgers, Newark, NJ, USA	3
*Aspergillus fumigatus* SRRC51	Human chest cavity lining	2
*Aspergillus fumigatus* SRRC1592	Rain forest soil	1

* *The most toxic strains are labeled with high values. All the strains are ranked based on their VOCs toxicities of the eclosion adult flies on the 10th and 15th days at 37°C*.

### Exposure of *Drosophila* 3rd Instar Larvae to VOCs Produced by Fungi

**Figure 1:**
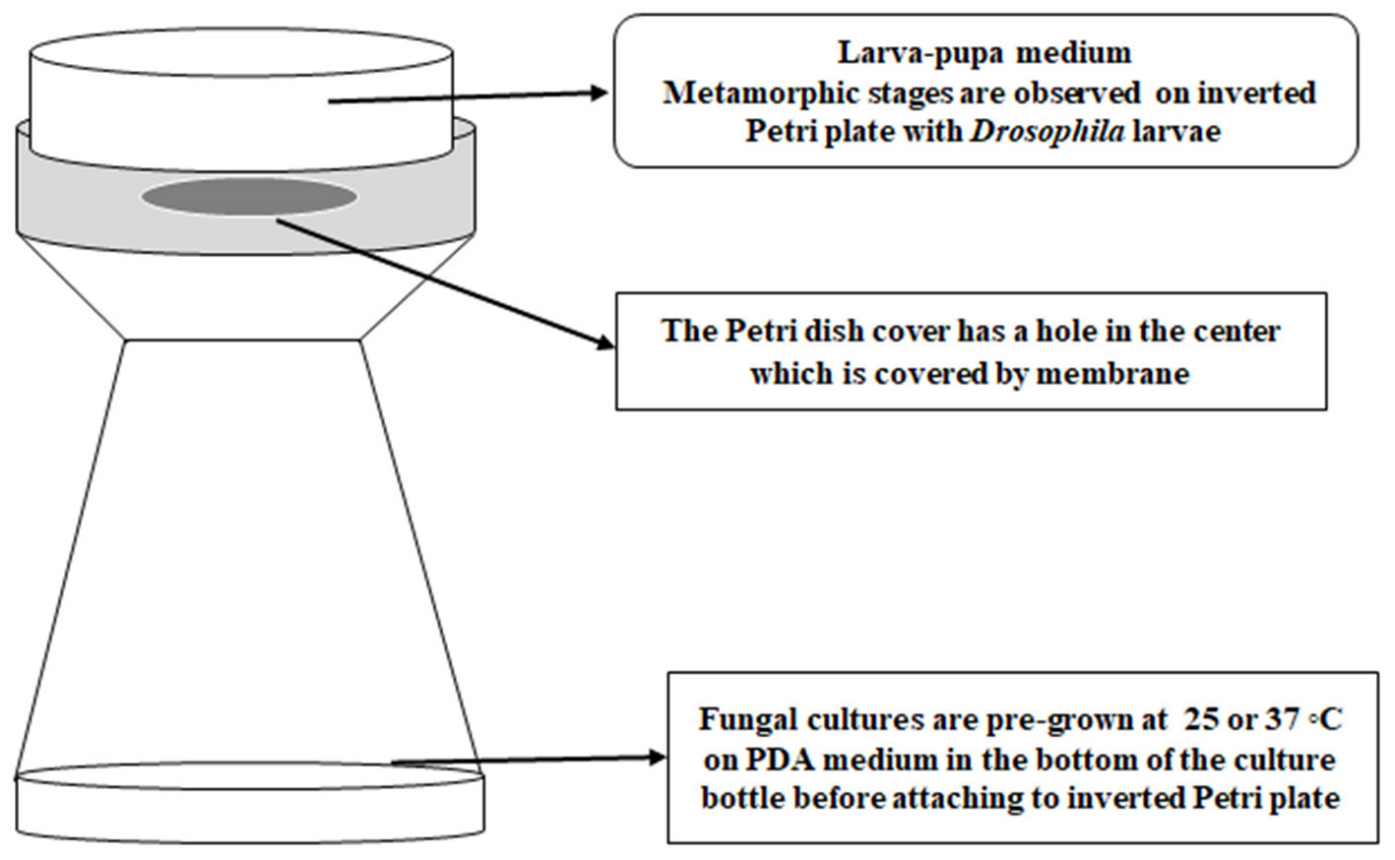
The bottle-plate microhabitat for placing *Drosophila* developmental stages and fungal cultures in a shared atmosphere without physical contact between the flies and fungi.

White-eyed *Drosophila* flies (*w*^1, 118^; *y*^1^) with a wild type immune system were maintained in Ward's Instant *Drosophila* medium (WARD's Natural Science, NY) before exposure studies with 3rd stage of instar larvae. Third instar fly larvae were exposed to VOCs following a modified version of the method of Inamdar et al. (2012). Instead of using a double-Petri plate set up, a *Drosophila* bottle with fungal cultures was used and attached via masking tape to a Petri plate cover that had been punctured with a flame-hot pipe to make a tri-circular opening and paired with a Petri plate of larval medium. Fifteen larvae were placed onto each larva-pupa medium plate, so that there were 15 larvae present in each “bottle-plate” microhabitat. Each Petri plate was secured on top of the bottle with a strip of clear tape across the edges of the plate (Figure 1). Different fungal strains pre-grown at 25°C for 5 days or at 37°C for 3 days were placed in the microhabitat with fifteen 3rd instar larvae, with three replicates for each temperature. The experiment was conducted in duplicate for a total of six bottles of larvae per strain and temperature. Control larvae were exposed to PDA medium without any fungi. All the bottle-plate microhabitats were incubated at 25°C with rotation at 50 rpm for 15 days. The numbers of larvae, pupae, and adult flies were counted daily. The differences in metamorphic stages and eclosion between controls and VOC-exposed strains were analyzed for significance by using Student *t*-test on the 4th day for the larvae, the 8th day for the pupal stage, and the 15th day for the adult flies.

### Gas Chromatography-Mass Spectrometry Analysis of *A. fumigatus* Volatiles

The VOCs from the most toxic (*A. fumigatus* SRRC 1607) and the least toxic (*A. fumigatus* SRRC 1592) strains were analyzed using Purge and Trap-Thermal Desorption-GC-MS (Hung et al., 2013; Zhao et al., 2017). Sterile PDA media and a blank containing only air were used as the negative controls. The fungi were grown either at 25°C for 5 days or at 37°C for 3 days on PDA in 250 mL flasks closed with plastic stoppers and sealed with Parafilm. Compounds were identified by comparison of spectra obtained from the *Aspergillus* samples with those from the reference library (NIST 08). Three replicates were performed for each fungal strain. The Tenax traps were spiked with 1.0 μg of benzene-d6, toluene-d8, and naphthalene-d8 as the internal standards.

## Results

### Effects of VOCs Produced by *Aspergillus fumigatus* Strains on *Drosophila* Metamorphosis

**Figure 2:**
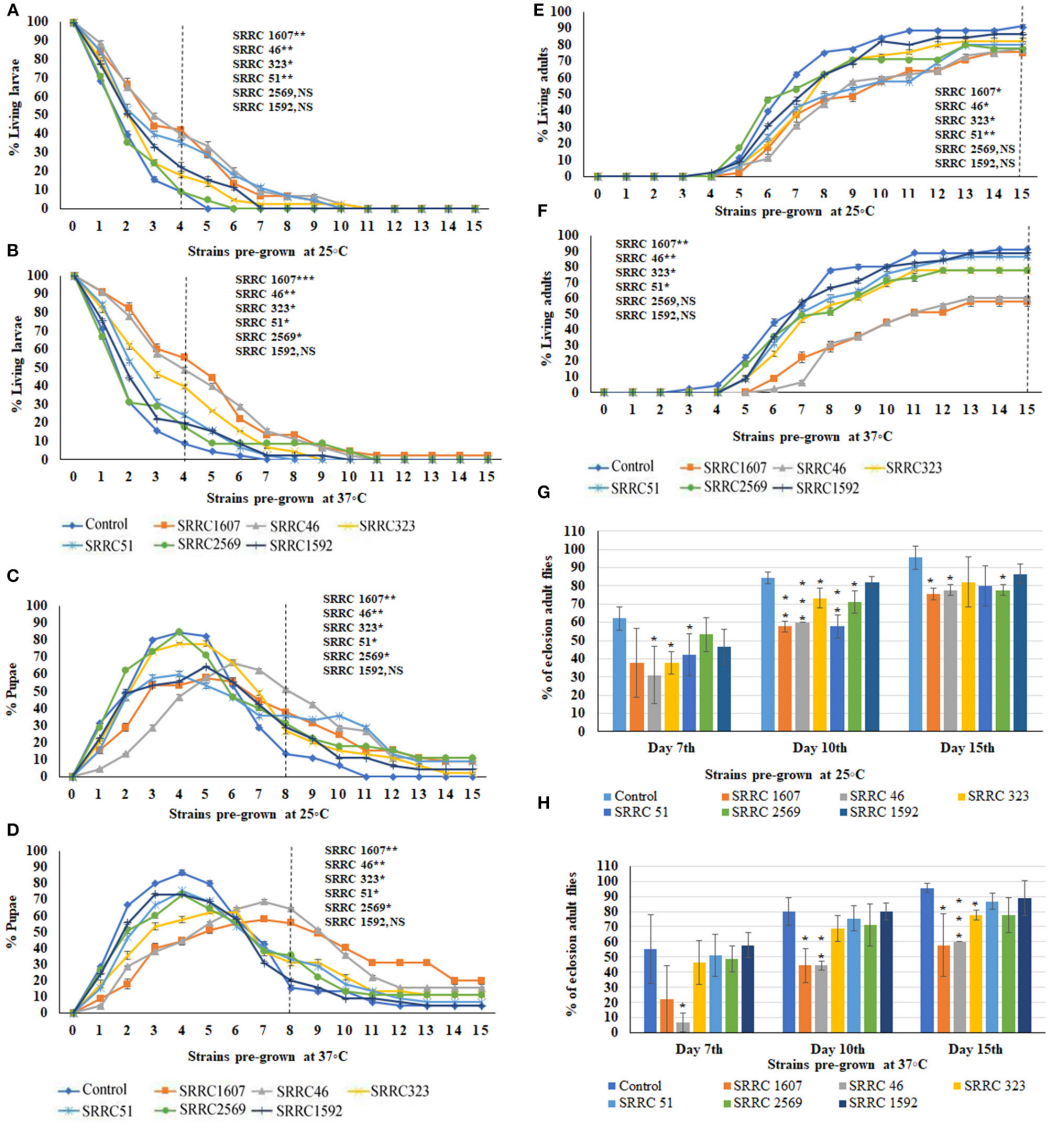
Percent living larval, pupal, and adult stages of 15 days of continuous exposure to VOCs emitted by six strains of *A. fumigatus*. The numbers of VOC-exposed larvae were compared to controls and the statistical differences were calculated on the 4th day: **(A)** Fungi were pre-grown at 25°C, **(B)** Fungi were pre-grown at 37°C. Where *represents the significant difference between controls and *A. fumigatus* exposed flies (*P < 0.05, **P < 0.005, ***P < 0.0005 and NS = Non-significant). A dashed vertical line denotes the day for statistical analysis. The numbers of VOC-exposed pupae were compared to controls and the statistical differences were calculated on the 8th day: **(C)** Fungi were pre-grown at 25°C, **(D)** Fungi were pre-grown at 37°C. The numbers of VOC-exposed adults were compared to controls and the statistical differences were calculated on the 15th day: **(E)** Fungi were pre-grown at 25°C; **(F)** Fungi were pre-grown at 37°C. Comparative analysis of percent of eclosion adult flies treated by VOCs emitted from six different strains of *A. fumigatus* (N = 90) at 7th, 10th, and 15th days at either 25°C **(G)** or 37°C **(H)**.

To evaluate the effects of VOCs emitted from six *A. fumigatus* strains, the numbers of larvae (Figures 2A,B), pupae (Figures 2C,D), and adults (Figures 2E,F) were counted daily for 15 days. The data in Figures 2A,C,E, show percentages of larval, pupal, and adult stages over 15 days when exposed to fungal cultures that had previously been grown at 25°C; the data in Figures 2B,D,F show the different metamorphic stages when the fungi had previously been grown at 37°C. Note, however, that in all cases, the 15-day exposure period of flies to VOCs was conducted at 25°C. Data are calculated as percentages of a given metamorphic stage during the *Drosophila* metamorphic cycle; thus, as numbers of larvae go down, the numbers of pupae increase. Similarly, numbers of pupae decrease when the flies eclose into adults. Some flies never eclose into adults, which reflects the percent toxicity from exposure to the VOCs (Figures 2G,H).

Normally, the larvae have pupated after 4 days and indeed, for control larvae, over 90% had pupated at this time. Significant delays in pupation were observed for all VOCs-exposed flies, with the most significant delays for larvae exposed to VOCs from *A. fumigatus* SRRC 46 and SRRC 1607, with fewer significant delays for larvae exposed to the VOCs released by *A. fumigatus* strains SRRC 2569 and 1592. On the 8th day, these delays were similar for fungi pre-grown at either temperature (Figures 2C,D). For example, nearly 38 and 56% of total flies remained in the pupal stage when exposed to VOCs emitted from *A. fumigatus* strain SRRC 1607 pre-grown at 25 and 37°C, respectively; however, only 13 and 16% were in the pupal stage for control treatments (Figures 2C,D).

The pupal stage usually lasts 3.5–4.5 days and then flies eclose into adults. Indeed, by the 10th day for the controls, 80–84% of the pupae had emerged as adults. However, for VOC-exposed flies, significant developmental delays were seen for the length of time spent in the pupal stage (Figures 2E,F). The numbers of adult flies on the 7th, 10th, and 15th days were compared and shown in Figures 2G,H. For strains SRRC 1607 and SRRC 46 pre-grown at 37°C, 44% had eclosed on the 10th day; only 58 and 60%, respectively, had eclosed on the 15th day; however, the larvae exposed to the VOCs released by SRRC 51 and SRRC 1592 had over 86% eclosion into adults; for strains SRRC 323 and 2569, eclosion rates were about 78% on the 15th day (Figure 2H). Overall, the toxic effects of *A. fumigatus* VOCs on the developmental stages of fruit flies were more significant when the fungi originally grew at 37°C than grew at 25°C. Exposure to a common atmosphere with VOCs from *A. fumigatus* strains SRRC 1607 and 46 significantly delayed the metamorphosis more than those exposed to VOCs from all the other strains (SRRC 51, 323, 2569, and 1952) which showed fewer significant differences compared to control treatments. Exposure to a common atmosphere with strain SRRC 1607 also yielded the most delays in metamorphosis and imposed the highest level of toxicity (Figure 2).

In order to determine if the greater toxicity seen with fungi pre-grown at 37°C was due to higher biomass, the six strains of *A. fumigatus* were measured for the dry weight cultivated at different temperatures. There were almost no differences in biomass at the two different temperatures (37 vs. 25°C) for any of the strains; however, SRRC 1607, which emitted the most toxic VOCs, had a lower biomass than the other five strains (Supplementary Figure 1).

### Effects of VOCs Emitted From *Candida albicans, Cryptococcus neoformans, Cryptococcus gattii*, and *Saccharomyces cerevisiae*

**Figure 3:**
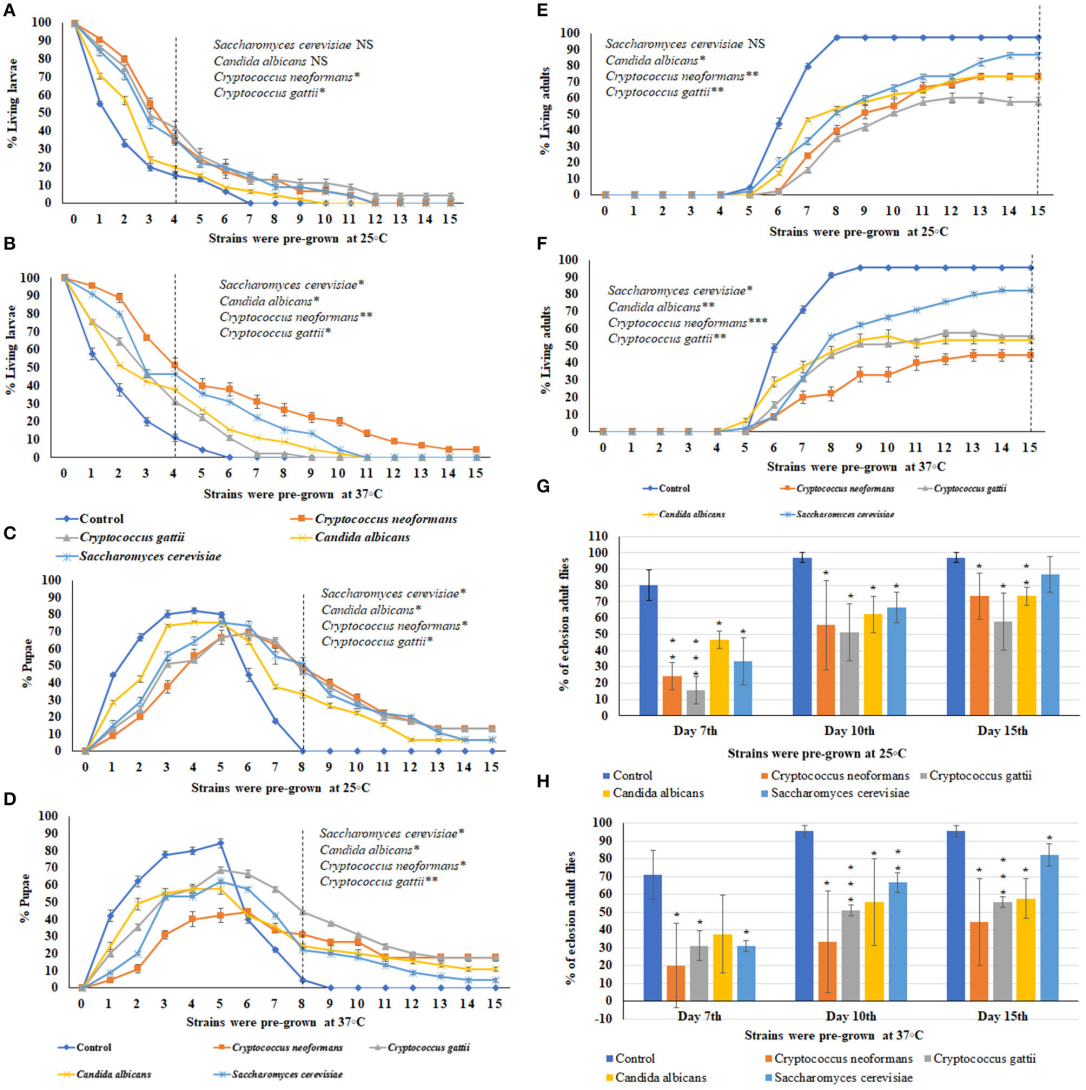
Percent larval, pupal, and adults of 15 days of continuous exposure to VOCs emitted by three yeast species and one *S. cerevisiae* strain. The numbers of VOC-exposed larvae were compared to controls and the statistical differences were calculated on the 4th day: **(A)** different strains were pre-grown at 25°C, **(B)** different strains were pre-grown at 37°C. Where *represents the significant difference between controls and VOC-exposed flies (*P < 0.05, **P < 0.005, ***P < 0.0005 and NS = Non-significant). A dashed vertical line denotes the day for statistical analysis. The numbers of VOC-exposed pupae were compared to controls and the statistical differences were calculated on the 8th day: **(C)** different strains were pre-grown at 25°C, **(D)** different strains were pre-grown at 37°C. The numbers of VOC-exposed adults were compared to controls and the statistical differences were calculated on the 15th day: **(E)** different strains were pre-grown at 25°C, (**F)** different strains were pre-grown at 37°C. Comparative analysis of percent of eclosion adult flies treated by VOCs emitted by three yeast species and one *S. cerevisiae* strain (N = 90) grown at 7th, 10th, and 15th days at either 25°C **(G)** or 37°C **(H)**.

Using the ecolosion assay to evaluate the effects of VOCs emitted from three medically important yeast strains and one *S. cerevisiae* strain, the numbers of larvae (Figures 3A,B), pupae (Figures 3C,D), and adults (Figures 3E,F) were counted daily for 15 days. The eclosion adult fly rates on the 7th, 10th, and 15th days are shown in Figures 3G,H. For sterile PDA media as the control, 85% of larvae had pupated by the 4th day and 95.5–97% of flies completed metamorphosis and eclosed by the 10th day (**Figures C,D,G,H**). For *S. cerevisiae* as a biological control, on the 10th day, the temperature of pre-incubation (25 or 37°C) was associated with no significant differences in the timing of metamorphosis and 67% of flies had eclosed into adults. On the 15th day, the eclosion adult rate was 87% for *S. cerevisiae* pre-grown at 25°C and 82% for those pre-grown at 37°C (Figures 3G,H). In contrast, the three medically important yeast species all emitted VOCs that caused delays in metamorphosis as well as toxicity. Delays in metamorphosis were observed for flies exposed to yeasts pre-grown at both 25 and 37°C; however, the delays were greater for larvae exposed to VOCs from yeasts pre-grown at the higher temperature. On the 4th day, 85% of controls had pupated; however, the percent pupation was 69, 61.2, 53.3, and 48.9% for those grown in a common atmosphere with VOCs emitted from *Cryptococcus gattii, Candida albicans, S. cerevisiae*, and *Cryptococcus neoformans*, respectively, pre-grown at 37°C (Figures 3A,B).

For *Candida albicans*, eclosion rates were 62.2 and 55.5% when exposed to VOCs from yeast cultures pre-grown at 25°C and at 37°C, respectively. For *Cryptococcus gattii*, the eclosion rates were 51.1% at both temperatures. For *C. neoformans*, the eclosion rates were 55.5 and 33.3% when exposed to VOCs from cultures pre-grown at 25°C and at 37°C, respectively. On the 15th day, for yeast strains pre-grown at 25°C, the eclosion rates were 55.7% for *Cryptococcus gattii*, and 73.3% for both *Candida albicans* and *Cryptococcus neoformans* (Figures 3E–H). On the 15th day for yeast strains pre-grown at 37°C, the eclosion rates were 57.7% for *Candia albicans*, 55.5% for *Cryptococcus gattii*, and 44.4% for *Cryptococcus neoformans* (Figures 3G,H). Overall, the flies exhibited more toxic effects and a longer delay in metamorphosis when exposed to the yeast strains pre-grown at 37°C than at 25°C.

### Comparative VOCs Toxicity From Different Strains and Morphology of VOCs Exposed Flies

Comparisons among VOCs emitted from *A. fumigatus* strains and *Candida albicans, Cryptococcus neoformans, Cryptococcus gattii*, and *Saccharomyces cerevisiae* pre-grown at 37°C were performed based on the 10th and 15th days of the eclosion rates and the toxicity rankings were listed in Table 1. The most toxic strains are *Cryptococcus* spp. and *A. fumigatus* SRRC1607, and their eclosion rates are below 58%; the least toxic strains are *Saccharomyces cerevisiae* and *A. fumigatus* SRRC1592 and their eclosion rates reach over 82% (Figures 2, 3 and Table 1).

We also observed certain morphological abnormalities in VOC-exposed *Drosophila* larval, pupal, and adult stages compared to control treatments. Dead larvae and pupae displayed dark pigmentation. VOC-exposed adults had wing and leg abnormalities. These morphological abnormalities were more pronounced when fly developmental stages were exposed to VOCs emitted from *A. fumigatus* than with the tested yeasts (Supplementary Figure 2).

### Purge and Trap-Thermal Desorption-GC-MS

**Figure 4:**
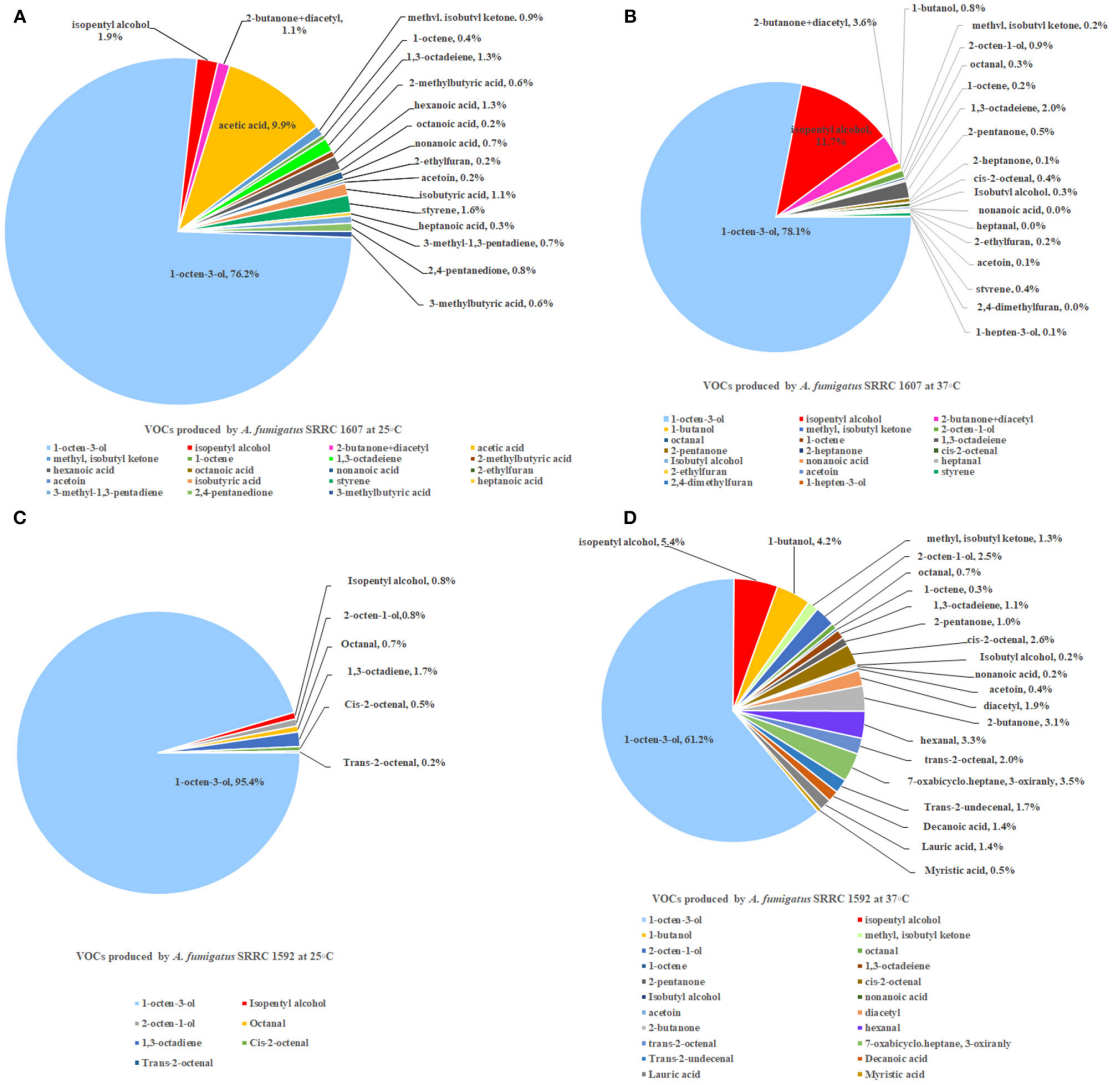
GC-MS analyses of VOCs from *A. fumigatus* strains SRRC 1607 and 1592. **(A)** VOCs emitted by SRRC 1607 cultivated for 5 day at 25°C. **(B)** VOCs emitted by SRRC 1607 cultivated for 3 day at 37°C. **(C)** VOCs emitted by SRRC 1592 cultivated for 5 day at 25°C. **(D)** VOCs emitted by SRRC 1592 cultivated for 3 days at 37°C.

Two strains of *A. fumigatus*, the most toxic SRRC 1607 strain and the least toxic SRRC 1592 strain, were selected for GC-MS analysis. These data were shown in Supplementary Table 1. Each strain had its own profile of VOCs at different temperatures. Both *A. fumigatus* strains produced 1-octen-3-ol, isopentyl alcohol, 1,3-octadeiene. *A. fumigatus* SRRC 1607 released higher amounts of different VOCs when it was pre-grown at 37°C (1978.8 ng) than at 25°C (684.5 ng). The most abundant VOC detected from both strains was 1-octen-3-ol, which was 521.4 ng (76.2%) at 25°C and 1544.7 ng (78.1%) at 37°C for the most toxigenic SRRC 1607 strain, and 164.5 ng (95.4%) at 25°C and 384 ng (61.2%) at 37°C for the least toxigenic SRRC 1592 strain (Figure 4 and Supplementary Table 1). SRRC 1607 also produced high amounts of isopentyl alcohol (13.3 ng, 1.9%) and acetic acid (67.7 ng, 9.9%) at 25°C; at 37°C, it produced 231.7 ng of isopentyl alcohol (11.7%) and 39.8 ng of 1,3-octadeiene (2.0%). Similarly, SRRC 1592 also produced high amounts of isopentyl alcohol (33.8 ng, 5.4%) and 1-butanol (26.4 ng, 4.2%); moreover, it produced more different VOCs including diacetyl, 7-oxabicyclo.heptane, 3-oxiranly, trans-2-undecenal, decanoic acid, lauric acid, and myristic acid (Figure 4 and Supplementary Table 1).

## Discussion

Filamentous fungi do not normally produce infections in healthy people and of the many species of known fungi, only a few cause diseases in humans and other animals. However, when patients are immunocompromised, opportunistic species including *A. fumigatus, Candida albicans, Cryptococcus gattii*, and *Cryptococcus neoformans* can cause infections. Modern medical practice has increased the number of immunocompromised patients and, concomitantly, fungal diseases such as invasive aspergillosis, invasive candidiasis, and cryptococcosis have become a more important cause of human mortality (Chamilos et al., 2007; Latgé and Steinbach, 2009; Casadevall, 2018; Almeida et al., 2019). Here, we hypothesized that the VOCs emitted by medically important fungi might be contributing to the virulence of opportunistic fungal infections and used a *Drosophila* model to test our hypothesis.

Past research with *Drosophila* models in medical mycology has utilized toll deficient mutant flies and infection by direct contact between the fungal pathogens and the flies (Alarco et al., 2004; Apidianakis et al., 2004; Chamilos et al., 2007; Hamilos et al., 2012; Lionakis and Kontoyiannis, 2012). In our studies, we used an eclosion assay. We placed third instar larvae from a white-eyed *Drosophila* strain with a wild-type immune system into a shared atmosphere with fungi that had been pre-grown at either 25 or 37°C such that there was no physical contact between the fungi and the flies. The numbers of larva, pupa, and adult flies were counted over 15 days of exposure to VOCs from six strains of *A. fumigatus*, and the pathogenic yeasts *Cryptococcus neoformans, Cryptococcus gattii*, and *Candida albicans*. The non-pathogenic yeast *S. cerevisiae* served as a biological control. In the eclosion assay, larvae exposed to VOCs from *S. cerevisiae* showed some delays in metamorphosis, but after 15 days, 82–87% of the flies nevertheless had eclosed into adults. In contrast, exposure to fungal VOCs from human pathogens delayed the time it took larvae to encapsulate into pupae, and then once at the pupal stage, delayed the time it took for pupae to eclose into adult flies. These effects were more pronounced when the fungal strains were pre-grown at 37°C, the temperature at which human infections occur, than when pre-grown at 25°C. For the *Aspergillus* strains, strain SRRC 1607 pre-grown at 37°C caused the greatest developmental delays, as well as displaying the highest mortality rate compared to the other *Aspergillus* strains. In a preliminary report using the *Drosophila* eclosion assay, we found similar toxicity of *A. fumigatus* strain SRRC 1607 VOCs to *Drosophila* larvae and hypothesized that volatiles might serve as virulence factors in aspergillosis (Al-Maliki et al., 2017). Furthermore, in previous research using a somewhat different version of this bioassay, we have showed that VOCs emitted by fungi isolated after hurricane events, and by chemical standards of 1-octen-3-ol and other eight carbon volatiles, caused toxicity and death in *Drosophila* larvae and adults (Inamdar et al., 2013; Inamdar and Bennett, 2014; Yin et al., 2015; Zhao et al., 2017). When several oxylipin volatiles were tested for their effects on *Drosophila* metamorphosis, body color of larvae and pupae became darker in the presence of 1-octen-3-ol and adult flies had abnormal wings (Yin et al., 2015). Here, similar morphological changes were observed when fly larvae were exposed to VOCs emitted by growing *A. fumigatus* cultures but not by exposure to VOCs emitted by the yeast species tested.

GC-MS analysis on the VOCs produced by the most toxic and the least toxic *A. fumigatus* strains was performed. Each strain produced a different VOCs signature; however, the single most abundant VOC detected was 1-octen-3-ol (Figure 4 and Supplementary Table 1). Other abundant compounds emitted by *A. fumigatus* strains pre-grown at 37°C included 2-butanone +diacetyl, 1,3-octadeiene, 2-octen-1-ol, isopentyl alcohol, and isobutyl alcohol. Many previous studies on the composition of fungal VOC mixtures show that 1-octen-3-ol is usually the single most abundant VOCs produced by molds and mushrooms (Combet et al., 2006). Moreover, in the past research, 1-octen-3-ol has been associated with numerous interspecific physiological effects. For example, in a study of filamentous fungi isolated after Hurricane Sandy, *Aspergillus niger* was the single most toxic mold. By solid-phase micro extraction-gas chromatography-mass spectrometry (SPME) analysis, high concentrations of 1-octen-3-ol were also found from this strain (Zhao et al., 2017). This aliphatic eight carbon alcohol is a degradation product of linoleic acid and is known to inhibit spore germination and growth in several fungal species (Chitarra et al., 2005; Combet et al., 2006; Noble et al., 2009; Herrero-Garcia et al., 2011; Berendsen et al., 2013). In animal systems, it is the natural ligand of bovine odorant-binding protein (Ramoni et al., 2001) and there is evidence that it is highly neurotoxic in human tissue culture lines as well as the cause of movement disorders in fruit flies (Inamdar et al., 2013). Furthermore, in human embryonic stem cells, 1-octen-3-ol is eighty times more toxic than toluene (Inamdar et al., 2012). When tested for fumigant properties, chemical standards of 1-octen-3-ol limit colony growth in *Saccharomyces cerevisiae* (Morath et al., 2017) and in *Pseudogymnoascus destructans*, the causative agent of white nose syndrome, it also inhibits mycelial growth (Padhi et al., 2017). In a transcriptome study on the effect of 1-octen-3-ol on *Penicillium chrysogenum*, genes for transporters and other membrane constituents were enriched, indicating that 1-octen-3-ol may be involved in membrane trafficking in fungi (Yin et al., 2019). Moreover, in recent work using a *Drosophila* model, Macedo et al. showed that mitochondria were a crucial target of 1-octen-3-ol toxicity, and caused alterations in levels of antioxidant enzymes, electron transport chain inhibition and apoptosis (Macedo et al., 2020). Using the yeast *Saccharomyces cerevisiae* as a host, a research group cloned the cDNA genes of lipoxygenase and hydroperoxide lyase from *Tricholoma matsutake* and biosynthesized (R)-(-)-1-octen-3-ol (Lee et al., 2020).

It is generally believed that pathogenic fungi possess virulence factors that allow them to grow in animals and cause diseases. Current known virulence factors include the ability to grow at 37°C, the capacity to evade host defenses, and the production of various toxins, adhesion factors or other metabolites that can cause host damages (Calderone and Clancy, 2011; Brunke et al., 2016). We suggest that VOCs emitted by pathogenic species of fungi may also serve as virulence factors. Scientists who study “sick building syndrome” have shown that there is a possible association between the presence of fungal VOCs and individuals who experience negative health effects when exposed to damp and moldy indoor environments (Mølhave, 2009). Toxicity depends on the chemical nature of the VOC and the level and length of exposures (Morath et al., 2012; Bennett and Inamdar, 2015). In one Brazilian study, colonies of *A. fumigatus, C. albicans, C. neoformans*, and *C. gattii* could be sub-cultivated from hospital air. When VOCs were assayed from this air, 1-pentanol, 1-octen-3-ol, 3-methyl-1-butanol, 3-octanol and 2-methyl-1-butanol were found in low concentrations while 2-heptanone and 2-methyl-1-propanol were present in high concentrations (Pantoja et al., 2016). In our study, *Cryptococcus* spp showed higher toxicity than *A. fumigatus* strain SRRC 1607 while the latter imposed more pronounced wing and leg abnormalities in flies than the former. The VOCs from *Cryptococcus* spp and the underlying mechanism that caused the differences will be investigated in our future experiments.

In summary, the data reported here demonstrate that in a *Drosophila* toxicity test, the VOCs emitted by growing cultures of *A. fumigatus, Candida albicans, Cryptococcus gattii*, and *Cryptococcus neoformans* cause significant delays in metamorphosis, along with significant lethality. In future research, it will be essential to determine which individual compounds such as 1-octen-3-ol, isopentyl alcohol, 1-butanol, in the VOC mixtures are causing the toxic effects and are therefore related to pathogenicity. VOCs from human pathogenic fungi, especially 1-octen-3-ol, may be previously unrecognized virulence factors that enhance the ability of these fungi to cause human disease. The *Drosophila* eclosion model provides a powerful reductionist method for studying the impact of fungal VOCs not only with respect to indoor air contamination, but also for shedding new light on factors that may enhance fungal pathogenesis and contribute to the differential virulence of medically important fungi. In future research, it will be important to analyze the VOCs emitted by pathogenic and non-pathogenic yeasts, as well as to use mammalian models to corroborate these data about the negative impact of fungal volatile compounds in certain fungal-animal interactions.

## Data Availability Statement

The original contributions presented in the study are included in the article/Supplementary Material, further inquiries can be directed to the corresponding author/s.

## Author Contributions

HA drafted the manuscript. AA and NG helped do some of the experiments. HA, GY, and JB discussed and revised the whole manuscript. All authors contributed to the article and approved the submitted version.

## Conflict of Interest

The authors declare that the research was conducted in the absence of any commercial or financial relationships that could be construed as a potential conflict of interest.

## Supplementary Material

Supplementary Figure 1 Dry weights of six *A. fumigatus* strains grown on potato dextrose broth at 25°C for 5 days or at 37°C for 3 days. There were no significant differences in dry weights between pairs of individual strains grown at the different temperatures; however, there was a significant difference in amount of growth between *A. fumigatus* strain SRRC 1607 and the other five strains (**P* < 0.05).

Supplementary Figure 2 The morphological effects of *Drosophila* exposed to VOCs from different strains. **(A)** larvae exposed to VOCs from control treatment for 4 days. **(B)** larvae exposed to VOCs from *Candida albicans* and *Cryptococcus* spp for 4 days. **(C)** larvae exposed VOCs from *A. fumigatus* strains for 10 days. **(D)** pupae exposed to VOCs from control treatments for 15 days. **(E)** pupae exposed to VOCs from *Candida albicans* and *Cryptococcus* spp for 15 days. **(F)** pupae exposed to VOCs from *A. fumigatus* strains for 15 days. **(G)** flies exposed to VOCs from control treatments for 15 days. **(H)** flies exposed to VOCs from *Candida albicans* and *Cryptococcus* spp for 15 days **(I)** flies exposed to VOCs from *A. fumigatus* strains for 15 days. The red arrows highlight the morphological abnormalities of larvae, pupae, and adults affected by VOCs from *A. fumigatus* strains.

Supplementary Table 1 Major VOCs produced by two *A. fumigatus* strains (SRRC 1607 and 1592) grown at either 25 or 37°C.
