# Supplementary figures and images for: Volatile Organic Compounds Produced by Human Pathogenic Fungi Are Toxic to Drosophila melanogaster
Source: Front Fungal Biol. 2021 Jan 18;1:629510. doi: 10.3389/ffunb.2020.629510 (PMC10512272; doi:10.3389/ffunb.2020.629510)

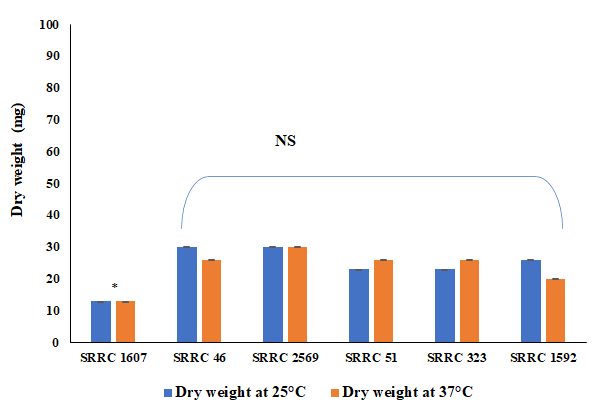

Supplement: Supplementary Figure 1 — Dry weights of six A. fumigatus strains grown on potato dextrose broth at 25°C for 5 days or at 37°C for 3 days. There were no significant differences in dry weights between pairs of individual strains grown at the different temperatures; however, there was a significant difference in amount of growth between A. fumigatus strain SRRC 1607 and the other five strains (*P < 0.05). [file Image_1.tif]

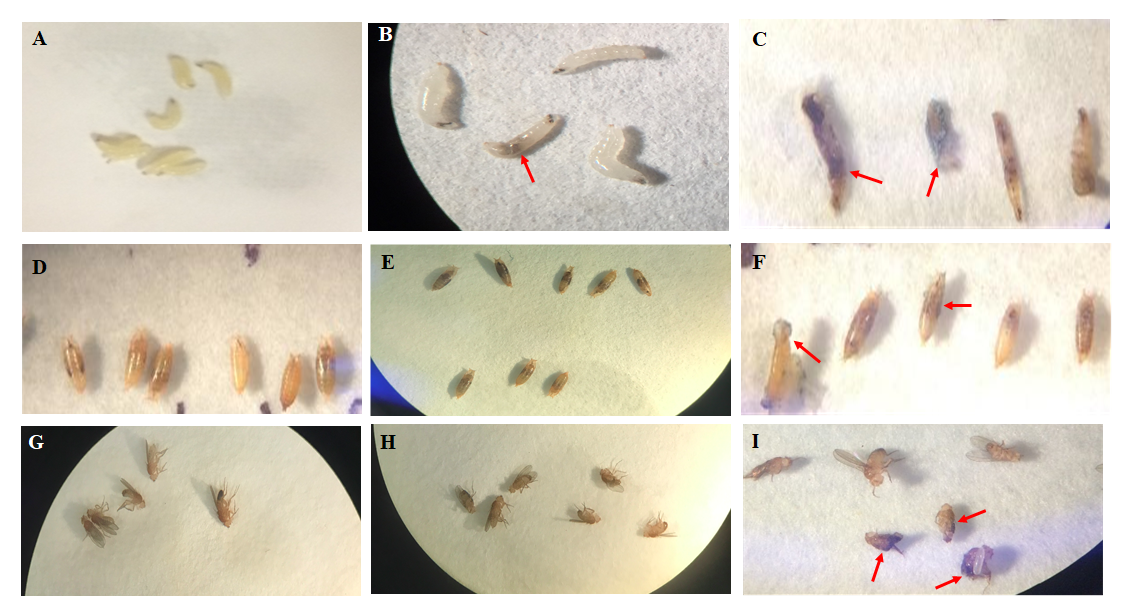

Supplement: Supplementary Figure 2 — The morphological effects of Drosophila exposed to VOCs from different strains. (A) larvae exposed to VOCs from control treatment for 4 days. (B) larvae exposed to VOCs from Candida albicans and Cryptococcus spp for 4 days. (C) larvae exposed VOCs from A. fumigatus strains for 10 days. (D) pupae exposed to VOCs from control treatments for 15 days. (E) pupae exposed to VOCs from Candida albicans and Cryptococcus spp for 15 days. (F) pupae exposed to VOCs from A. fumigatus strains for 15 days. (G) flies exposed to VOCs from control treatments for 15 days. (H) flies exposed to VOCs from Candida albicans and Cryptococcus spp for 15 days (I) flies exposed to VOCs from A. fumigatus strains for 15 days. The red arrows highlight the morphological abnormalities of larvae, pupae, and adults affected by VOCs from A. fumigatus strains. [file Image_2.tif]
