# Supplementary material for: Volatile Organic Compounds Produced by Human Pathogenic Fungi Are Toxic to Drosophila melanogaster
Source: Front Fungal Biol. 2021 Jan 18;1:629510. doi: 10.3389/ffunb.2020.629510 (PMC10512272; doi:10.3389/ffunb.2020.629510)
Supplement: Supplementary Table 1 — Major VOCs produced by two A. fumigatus strains (SRRC 1607 and 1592) grown at either 25 or 37°C. [file Table_1.docx]

**SUPPLEMENTARY TABLE 1** Major VOCs produced by two *A*. *fumigatus* strains (SRRC 1607 and 1592) grown at either 25^o^C or 37^o^C.

| Compounds (ng/trap) | SRRC 1607  25◦C 37◦C | | SRRC 1592  25◦C 37◦C | |
| --- | --- | --- | --- | --- |
| 1-octen-3-ol | 521.4 | 1544.7^a^ | 164.5 | 384^b^ |
| isopentyl alcohol | 13.3 | 231.7 | 1.3 | 33.8 |
| 2-butanone+diacetyl | 7.6 | 71.1 | nd | nd |
| 1-butanol | nd | 15.7 | nd | 26.4 |
| acetic acid | 67.7 | nd | nd | nd |
| methyl, isobutyl ketone | 6.3 | 3.3 | nd | 8.3 |
| 2-octen-1-ol | nd | 17.4 | 1.4 | 15.9 |
| octanal | nd | 5.8 | 1.2 | 4.5 |
| 1-octene | 3.0 | 4.2 | nd | 1.9 |
| 1,3-octadeiene | 8.7 | 39.8 | 2.9 | 6.9 |
| 2-methylbutyric acid | 3.8 | nd | nd | nd |
| hexanoic acid | 8.9 | nd | nd | nd |
| octanoic acid | 1.5 | nd | nd | nd |
| 2-pentanone | nd | 9.9 | nd | 6.5 |
| 2-heptanone | nd | 1.8 | nd | nd |
| cis-2-octenal | nd | 8.6 | 0.9 | 16 |
| Isobutyl alcohol | nd | 5.7 | nd | 1.4 |
| nonanoic acid | 4.8 | 0.9 | nd | 1.1 |
| heptanal | nd | 0.5 | nd | nd |
| 2-ethylfuran | 1.5 | 4.4 | nd | nd |
| acetoin | 1.5 | 2.4 | nd | 2.6 |
| isobutyric acid | 7.7 | nd | nd | nd |
| styrene | 10.9 | 8.8 | nd | nd |
| 2,4-dimethylfuran | nd | 1.0 | nd | nd |
| 1-hepten-3-ol | nd | 1.1 | nd | nd |
| heptanoic acid | 2.2 | nd | nd | nd |
| 3-methyl-1,3-pentadiene | 4.7 | nd | nd | nd |
| 2,4-pentanedione | 5.2 | nd | nd | nd |
| 3-methylbutyric acid | 3.8 | nd | nd | nd |
| Trans-2-octenal | nd | nd | 0.4 | 12.3 |
| diacetyl | nd | nd | nd | 12.1 |
| 2-butanone | nd | nd | nd | 19.4 |
| hexanal | nd | nd | nd | 20.7 |
| 7-oxabicyclo.heptane, 3-oxiranly | nd | nd | nd | 22.1 |
| Trans-2-undecenal | nd | nd | nd | 10.9 |
| Decanoic acid | nd | nd | nd | 8.5 |
| Lauric acid | nd | nd | nd | 9.0 |
| Myristic acid | nd | nd | nd | 3.1 |
| Total amount (ng) | 684.5 | 1978.8 | 172.6 | 627.4 |

^a^The most abundant compound was detected by the most toxic strain *A*. *fumigatus* SRRC 1607 in high concentration when the strain was pre-grown on at 37◦C than at 25◦C. ^b^The most abundant compound was detected by the least toxic strain SRRC 1592 in low concentration when the strain was pre-grown at both temperatures. The “nd” indicates not detected. Data are the mean values of two individual tests.
